# The Health of the Dentist

**Published:** 1874-04

**Authors:** 


### Editorial.

### THE HEALTH OF THE DENTIST.

This is a subject of very great importance to every member of our profession. It is one requiring more special attention by dentists than by many other classes of persons. Dental practice is an occupation not conducive to health. The confinement of the dentist, and that too often, in close and ill ventilated rooms is prejudicial to health. The want of pure air and light—direct sunlight—is a very prolific cause of disease, and in the selection and arrangement of an office particular attention should be paid to these.

With almost all operators the position of the body while operating is unnatural, cramped or constrained; important organs of the body are compressed and their proper function interrupted; this, if long continued, will produce difficulty and, too often, disease, more or less marked, according to the susceptibilities.

And, in addition to this, the inhalation of offensive, and sometimes poisonous, matter proceeding from diseased patients, is doubtless a fruitful source of the affections experienced by dentists.

Then, again, the psychic influence of incompatible patients upon highly sensitive persons is injurious in a very marked degree, not only increasing nervous irritability, but making definite impress upon the functions of the body.

Now, these four or five particulars will account for a large share of the particular affections to which dentists as a class are subject.

Many suffer enfeeblement from imperfect nutrition, this resulting from want of food. The system through sluggishness—a want of proper exercise—does not demand it, and will not kindly receive it, or, at least, will not appropriate it if taken. The dentist should take, every morning and evening at least, an amount of exercise to answer the demands of his system—some should have far more than others. This exercise should be such as to bring into play, so far as possible, every organ of the body; indeed, every tissue should be made to feel the influence of such exercise; and particularly, in every instance, should a free action of the skin be induced, not necessarily, however, to sensible perspiration. The great object in exercise is, to give every organ the utmost freedom of action, but without coercion.

The position of the body while operating is a subject to which especial attention should be given. Much injury is doubtless caused by the constrained and unnatural attitude assumed by many when operating. By throwing the head forward and stooping, and drawing the shoulders forward and together, as the custom of many is, diminishes the capacity of the chest and compresses the respiratory organs, so as to much abridge their full action. The lungs should always act freely and up to their capacity. No portion of this organism should be thrown into disuse, even for a short time; and can not be without injury. This stooping and twisted position assumed by some, together with compression upon the abdominal region occasioned by leaning against the chair, will operate injury upon the digestive apparatus; the compression produces interrupted digestive function and forces blood from one part and an undue amount of it, to another part or parts. The rectal portion of the colon is a very susceptible portion for such determination. It is very highly ramified over its internal surface with blood-vessels of considerable size. Owing to these and other conditions that attach to this structure, it often becomes the seat of disease exhausting in its effects and often serious in its results; an affection always annoying and frequently incapacitating the dentist for the proper performance of his duties.

The interruption or disturbance of nerve function and consequent nervous irritability is a very common affection of the dentist. There are many things that tend to produce this high objectionable condition. The high tension requisite in the performance of delicate and difficult operations upon the teeth, long continued; the exhausting labor involved: these, in connection with the depressing and exhausting influence of definite incompatibility on the part of the patient, are calculated to operate disastrously upon any one of a sensitive nature.

In reference to this whole subject we will now venture only a suggestion or two.

And, in the first place, the dentist should never work to extreme fatigue. Again, mental tension should not be too long sustained without relaxation. Usually a few minutes will be quite sufficient, unless there is much fatigue. The use of the rubber dam and the automatic saliva pump renders practicable short relaxations, as often as may be required; and no reasonable patient will object, when aware that such respites are to his own advantage as well as to that of the operator.

The operator should have all his arrangements, appliances and instruments of the most perfect kind and in the best possible condition, that everything may be done with the utmost ease and facility—with as little friction as possible. Do not permit annoyance and irritants to have a place about a dental office, but let everything be of the most cheerful, inviting and pleasing character.

No dentist can, with justice to himself or his patient, operate for one who has a manifest incompatibility, unless, by nerve energy and will power, he can completely dominate it or so hold his own resources that they can not be affected by it. No dentist who is feeble and out of health should attempt to operate. The dentist should make it one of his first objects, to maintain good health, vigorous energy, correct and commanding will force, clear perception and a well-balanced mental and physical being in every respect.

